# Safety and Antibody Response After 1 and 2 Doses of BNT162b2 mRNA Vaccine in Recipients of Allogeneic Hematopoietic Stem Cell Transplant

**DOI:** 10.1001/jamanetworkopen.2021.26344

**Published:** 2021-09-14

**Authors:** Amandine Le Bourgeois, Marianne Coste-Burel, Thierry Guillaume, Pierre Peterlin, Alice Garnier, Marie C. Béné, Patrice Chevallier

**Affiliations:** 1 Hematology Department, Nantes University Hospital, Nantes, France; 2 Virology Department, Nantes University Hospital, Nantes, France; 3 Institut National de al Santé et de la Recherche Médicale, Unité Mixte Recherche 1232, Centre de Recherche en Cancérologie et Immunologie Nantes Angers Institut de Recherche en Santé de l’Université de Nantes, University of Nantes, France; 4 Hematology Biology, Nantes University Hospital, Nantes, France

## Abstract

This cohort study examines safety and antibody responses after 1 and 2 doses of BNT162b2 mRNA vaccine in recipients of allogeneic hematopoietic stem cell transplant (HSCT).

## Introduction

COVID-19, which is due to infection with SARS-CoV-2, results in poor outcomes in patients with hematologic cancers (approximately 40% mortality rate).^1,2^ The efficacy of anti-SARS-CoV-2 mRNA vaccines has been successfully demonstrated in healthy populations^3^ and also has been reported in immunocompromised patients. Recently, we showed that a first injection of the BNT162b2 (Pfizer-BioNTech) vaccine induced an antibody response in 55% of 112 allogeneic hematopoietic stem cell transplant (HSCT) recipients.^4^ Here, we document the antibody response to a second dose of BNT162b2 vaccine in an extended cohort of 117 patients.

## Methods

This single-center cohort study enrolled allogeneic HSCT recipients with no clinical history of COVID-19 and no active graft-vs-host disease more than 3 months after transplant. Vaccination was performed in our department between January 20 and April 17, 2021. All participants provided written informed consent, and the study was approved by Nantes University Hospital review board. All procedures followed were in accordance with the ethical standards of the responsible committee on human experimentation (institutional and national) and with the Helsinki Declaration of 1975, as revised in 2008.

Previous asymptomatic COVID-19 infection was investigated before the first vaccine injection by testing for antinucleocapsid antibodies (anti–SARS-CoV-2 immunoassay; Roche Elecsys). Antibody responses to the SARS-CoV-2 spike protein receptor–binding domain (Elecsys anti–SARS-CoV-2-S) were tested twice, at the time of the second injection and approximately 1 month after the second injection. As recommended by the manufacturer, titers greater than or equal to 0.8 U/mL were considered positive, with the highest value being greater than 250 U/mL. Associations between clinical characteristics and antibody responses were investigated using 1-sided χ^2^ and Wilcoxon tests with R statistical software version 4.0.2 (R Project for Statistical Computing) via BiostaTGV. *P* < .05 was considered significant.

## Results

Previous asymptomatic SARS-CoV-2 infection was documented in 4 of 121 enrolled patients, who were, therefore, excluded from the study. They were vaccinated twice, and all reached specific IgG titers greater than 250 U/mL after the second dose. Characteristics of the 117 allogeneic HSCT recipients retained (median [range] age, 57 [20-75] years; 70 men [60%]) are provided in the Table. The median (range) interval between the first and the second dose was 22 (16-37) days. At the time of the second injection, 63 patients (54%) had a positive anti-spike antibody response. The median IgG titer for responders was 15.8 U/mL and ranged from 0.9 U/mL to more than 250 U/mL, with the latter occurring in 4 patients (3%). The second antibody testing was performed at a median (range) interval of 35 (18-77) days after the second dose and was positive in 97 patients (83%), with IgG titers ranging from 0.9 U/mL to greater than 250 U/mL; 72 (62%) patients reached the highest IgG titer. Factors associated with the absence of response were a haplotransplant, recent (<1 year) HSCT (Figure), lymphopenia (<1000 cells/μL; to convert lymphocyte count to cells × 10^9^/L, multiply by 0.001), and receipt of immunosuppressive treatment or chemotherapy at the time of vaccination (Table).

**Table.** Patient Characteristics and Results of Serological Tests After Dose 1 and Dose 2

Characteristics	Total, participants, No. (%) (N = 117)	Antibody response after 1 dose, No. (%) (n = 63)	*P* value	Antibody response after 2 doses, No. (%) (n = 97)	*P* value
Age, y					
Median (range)	57 (20-75)	NA	.30	NA	.47
<40	17 (14)	11 (65)		16 (94)	
40-59	50 (43)	29 (58)		41 (82)	
≥60	50 (43)	23 (46)		40 (80)	
Sex					
Male	70 (60)	34 (49)	.22	56 (80)	.44
Female	47 (40)	29 (62)		41 (87)	
Time from transplant to vaccination, mo					
Median (range), d	654 (91-6198)	NA		NA	
<12	29 (25)	4 (14)	.001	15 (52)	<.001
12-24	36 (31)	18 (50)		32 (89)	
>24	52 (44)	41 (79)		50 (96)	
Underlying disease^a^					
Myeloid	77 (68)	40 (52)	.87	73 (95)	.93
Lymphoid	36 (32)	20 (55)		30 (83)	
Donor type					
Genoidentical	30 (25.5)	16 (53)	.46	26 (87)	.03
Matched unrelated donor	49 (42)	28 (57)		44 (90)	
Haploidentical	36 (31)	17 (47)		25 (69)	
Mismatched unrelated donor (9/10 human leukocyte antigens)	2 (2)	2 (100)		2 (100)	
Conditioning					
Myeloablative	23 (20)	15 (65)	.44	22 (96)	.36
Reduced intensity	87 (74)	44 (56)		70 (80)	
Sequential	7 (6)	4 (57)		5 (71)	
Previous graft-vs-host disease					
Yes	62 (53)	32 (51)	.74	51 (82)	.87
No	55 (47)	31 (57)		46 (84)	
Ongoing treatment^b^					
No	85 (73)	53 (62)	.005	77 (915)	<.001^c^
Yes	32 (27)	10 (31)		20 (63)	
Corticosteroids^d^	13	NA	NA	NA	NA
Ruxolitinib	3	NA		NA	
Ciclosporin A	10	NA		NA	
Chemotherapy	6	NA		NA	
Lymphocyte count at vaccination					
Median (range), cells/μL	1400 (150-9880)	NA	<.001	NA	<.001
<1000 cell/μL (n = 36)	36 (31)	9 (25)		23 (64)	
≥1000 cells/μL (n = 81)	81 (69)	54 (67)		74 (91)	

Abbreviation: NA, not applicable.

SI conversion factor: To convert lymphocyte count to cells × 10^9^/L, multiply by 0.001.

^a^ Includes acute myeloblastic leukemia (37 patients), myelodysplastic syndrome (22 patients), myelofibrosis (8 patients), myelodysplastic syndrome and myelofibrosis (5 patients), chronic myelomonocytic leukemia (4 patients), blastic plasmacytoid dendritic cell neoplasm (1 patient), non-Hodgkin lymphoma (20 patients), Hodgkin lymphoma (4 patients), acute lymphoblastic leukemia (11 patients), multiple myeloma (1 patient), nonlymphoid and nonmyeloid aplastic anemia (3 patients), and porphyria (1 patient).

^b^ Includes immunosuppressive drugs or chemotherapy for relapse or relapse prevention.

^c^ *P* value compares genoidentical, matched unrelated, and haploidentical donors.

^d^ Refers to corticosteroids used alone or in combination (ruxolitinib, cyclosporin A, and mycophenolate mofetyl).

**Figure.**
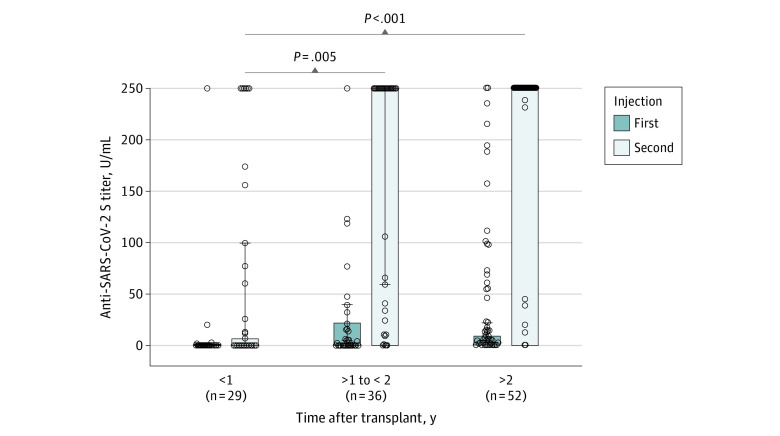
Anti–SARS-CoV-2 Titers After the First and Second Vaccine Injections Box plots show anti–SARS-CoV-2 spike protein receptor titers after the first and the second vaccine injections. Lines within boxes denote medians, error bars denote 95% CIs, circles denote data for individual patients. Comparisons are between patients for whom follow-up after allogeneic hematopoietic stem cell transplant was less than 1 year vs between 1 and 2 years vs more than 2 years.

Patients were requested to answer questionnaires for 7 days following dose 1 and dose 2. The responses showed that the 2 vaccine injections were very safe. Only grade 1 or 2 adverse reactions occurred in 51 of 106 patients (48%) after dose 1 and 34 of 87 patients (39%) after dose 2. These rates were comparable to those for a healthy vaccinated population of 25 caregivers from the hematology department of Nantes University Hospital, who all achieved the highest IgG titer after the second dose. Finally, at a median (range) follow-up of 58 (39-98) days, no COVID-19 infection was documented.

## Discussion

Despite the limitations inherent to an observational analysis and the fact that the cohort was small and from a single center, this study found a high response rate of 83% in this cohort of allogeneic HSCT recipients after 2 doses of BNT162b2 vaccine. Of note, 62% of the patients achieved the highest IgG titer also reached by a concomitant healthy cohort. This is much more than the 54% rate of seroconversion that has been reported after 2 doses in solid-organ transplant recipients^5^ and compares favorably with data obtained in patients treated for solid tumors, for whom a 95% of response rate was obtained after the second dose.^6^ This humoral response is, however, only 1 marker of immunity, and allogeneic HSCT recipients will likely have differences in T cell reactivity that should be explored.
